# Rare entrapment neuropathies of the lower extremity: A narrative review

**DOI:** 10.1097/MD.0000000000039486

**Published:** 2024-08-30

**Authors:** Nicu Cătălin Drăghici, Roxana Bolchis, Livia Livinț Popa, Vitalie Văcăraș, Silvina Iluț, Atamyrat Bashimov, Diana Maria Domnița, Hanna Maria Dragoș, Irina Vlad, Dafin Fior Mureșanu

**Affiliations:** a IMOGEN Institute, Centre of Advanced Research Studies, Cluj-Napoca, Romania; b RoNeuro Institute, Centre for Neurological Research and Diagnostic, Cluj-Napoca, Romania; c Department of Clinical Neurosciences, Iuliu Hațieganu University of Medicine and Pharmacy, Cluj-Napoca, Romania; d Faculty of Medicine, Iuliu Hațieganu University of Medicine and Pharmacy, Cluj-Napoca, Romania.

**Keywords:** Morton neuroma, nerve compression syndromes, obturator nerve, superior gluteal nerve entrapment, tarsal tunnel syndrome, tibial nerve, tibial neuropathy

## Abstract

Lower limb entrapment neuropathies comprise a wide range of disorders, including less common conditions like tarsal tunnel syndrome, Morton neuroma, obturator nerve entrapment syndrome, superior gluteal nerve entrapment, and cluneal nerve entrapment syndrome. Despite being less prevalent, these syndromes are equally significant, presenting with symptoms such as pain, dysesthesia, muscular weakness, and distinct physical signs. Accurate diagnosis of these less common disorders is crucial for successful therapy and patient recovery, as they can sometimes be mistaken for lumbar plexopathies, radiculopathies, or musculotendinous diseases. This narrative review highlights the significance of identifying and diagnosing these particular neuropathies through a comprehensive assessment of the patient’s medical history, detailed physical examination, and the use of electrodiagnostic and/or ultrasound investigations. When the diagnosis is uncertain, advanced imaging techniques like magnetic resonance neurography or magnetic resonance imaging are necessary to confirm the diagnosis. A positive diagnosis ensures prompt and targeted treatments, preventing further nerve impairments and muscle wasting. This article explores the epidemiology, anatomy, pathophysiology, etiology, clinical presentation, and electrodiagnostic interpretation of lower limb entrapment neuropathies, highlighting the importance of precise diagnosis in achieving favorable patient outcomes.

## 1. Introduction

Lower limb entrapment neuropathies are a category of condition that is frequently misdiagnosed and overlooked. This group includes uncommon disorders such as tarsal tunnel syndrome, Morton neuroma, obturator nerve entrapment syndrome, superior gluteal nerve entrapment, and cluneal nerve entrapment syndrome. Due to their complexity, delayed treatment often occurs, resulting in persistent pain and disabilities. The clinicians’ reluctance to diagnose these medical conditions derives from the absence of standardized diagnostic tests and explicit clinical criteria defined in the medical literature. This narrative review provides a comprehensive understanding of rarely encountered entrapment neuropathies, describing their anatomy, pathogenesis, clinical presentation, and electrodiagnostic (EDX) measurements in patients with this condition.

## 2. Materials and methods

A comprehensive literature review was conducted using the databases, Pubmed, Cochrane Library, Google Scholar, and EMBASE, including literature published between 1950 and 2024. The primary keywords used included “Entrapment Neuropathy,” “Lower Extremity,” “Peripheral Nerve Entrapment,” “Uncommon Neuropathies,” and “Rare Neuropathies.” These were complemented by secondary keywords such as “Diagnosis,” “Management,” “Treatment Outcomes,” “Electrodiagnostic Testing,” and “Clinical Presentation.” We used boolean operators to associate all keywords in order to identify articles that are directly related to our study.

The study design targeted includes peer-reviewed original research articles, systematic reviews, meta-analyses, and significant case reports, given the rarity of the conditions under discussion. Excluded were non-peer-reviewed articles, incomplete studies lacking clear methodologies or outcomes, and non-English articles.

This structured approach to data collection was instrumental in compiling a focused and thorough narrative review, elucidating the clinical presentations, diagnostic challenges, and management options for these less commonly encountered neuropathies in the lower extremity. The aim was to provide a detailed and insightful resource that would be valuable for clinicians and researchers alike, enhancing understanding and improving patient care outcomes for these rare neurological conditions.

## 3. Morton neuroma

### 3.1. Introduction

Intermetatarsal neuropathy frequently leads to foot pain and discomfort, particularly in the third metatarsal region.^[1]^ The exact incidence is still unclear, but the median age of diagnosis is 50 years. Furthermore, women are affected 10 times more than men.^[2]^ The disease was named in honor of Thomas G. Morton, the initial author, who provided a series of cases about this particular clinical condition.^[1,2]^

### 3.2. Anatomy, etiology, and pathophysiology

**Figure 1.**
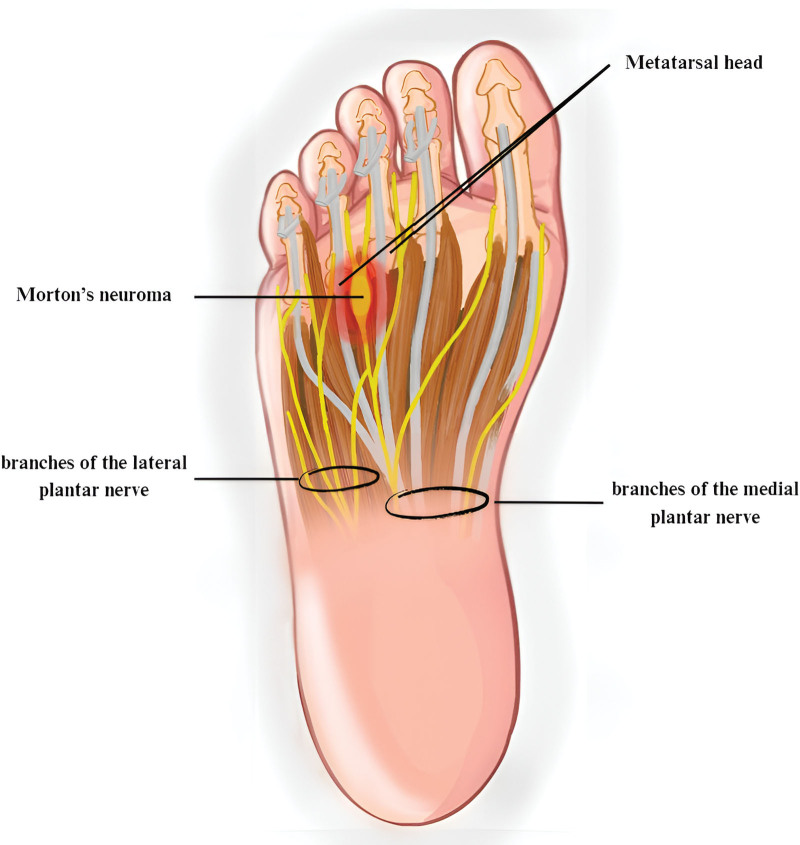
This illustration provides a detailed plantar perspective of Morton neuroma, showing the common digital nerve’s enlargement between the third and fourth metatarsal heads, where the neuroma most frequently occurs. The image illustrates the adjacent anatomical structures, including the metatarsal bones, intermetatarsal spaces, and adjacent soft tissues, highlighting the neuroma’s typical location and its impact on surrounding neural and vascular structures.

The plantar nerves are responsible for the sensory and muscular innervation of the fourth intermetatarsal space.^[3]^ The medial plantar nerve (MPN) divides into the proper plantar digital hallux nerve and the common plantar digital nerves I, II, and III. The lateral plantar nerve (LPN) splits into the proper plantar digital nerve of the V toe and the IV common plantar digital nerve.^[2,3]^ Furthermore, in more than 50% of cases, the LPN provides anastomotic branches to the III common digital plantar nerve. This makes the nerve bigger and more likely to get compressed.^[1,3]^ Additionally, the deep transverse metatarsal ligament (DTML) divides the intermetatarsal spaces into 2 horizontal compartments. The upper compartment contains the intermetatarsal synovial bursa, which is in direct contact with DTML. Likewise, the intermetatarsal synovial bursa covers the anterior edge of the DTML in the third and fourth intermetatarsal spaces, which have direct contact with the III and IV common plantar digital nerves^[1]^ (Fig. 1).

The thickening of a nerve, which is not neoplastic but rather the result of nerve degeneration and perineural fibrosis, is what defines the condition known as a neuroma.^[1,2,4]^ Four primary pathophysiological ideas could clarify the etiology of Morton neuroma.^[1,2]^ The dominant is the “chronic trauma theory,” which argues that there is persistent compressive damage on the common plantar nerves between the metatarsal heads and metatarsophalangeal joints when walking. The observation that the III and IV intermetatarsal spaces are narrower than other intermetatarsal spaces and are therefore the most common sites for Morton neuroma supports the theory. Therefore, 2/3 of cases are located on the III common plantar nerve.^[1,2]^ Additional theories include “entrapment theory,” “intermetatarsal bursitis theory,” and “ischemic theory.” Each theory may explain specific elements of this medical condition, and many researchers argue that these theories can coexist and are not mutually exclusive.^[1,2]^

### 3.3. Clinical presentation and physical examination

The typical person suffering from Morton neuroma is a middle-aged woman with a history of wearing high-heeled and/or narrow-toe shoes.^[1,4]^ The patients usually describe a specific burning sensation on the plantar surface of the foot, including in the area around the intermetatarsal space where the injured common plantar nerve is located. Paresthesia and a tingling sensation could accompany the pain and radiate to the corresponding toes.^[1,2,4]^ The patients additionally describe a subjective sensation of walking on a small, solid object, often described as a little stone, a folded sock, or a deformed shoe. The symptoms are exacerbated by using ill-fitting shoes and can be reduced by changing to more comfortable footwear and/or applying foot massages.^[1,2,5]^ In addition, clinicians have the option to use several provocative tests with positive findings when pain and/or paresthesia are induced. An intermetatarsal tenderness test can be conducted by applying pressure to the affected area.^[1,2,4]^ Another diagnostic is Tinel test, which involves tapping on the suspected intermetatarsal space.^[2]^ Nevertheless, the most accurate and precise test is Mulder test.^[3]^ To do this examination, physicians compress the foot with one hand and apply axial pressure to the suspected intermetatarsal area with the other hand. This procedure causes a sharp pain and an unusual tactile click.^[2–5]^ It is important to note that patients may have multilocal conditions in different locations on the same foot or be impacted bilaterally.^[2]^

### 3.4. Electrodiagnostic and imaging techniques

The diagnosis of Morton neuroma can be achieved by evaluating the patient’s clinical history followed by a physical examination, which has a high sensitivity rate of 98%.^[6]^ The provocative tests are highly useful for confirming the diagnosis, particularly Mulder test, which has a sensitivity of 94% to 98%.^[3]^

**Figure 2.**
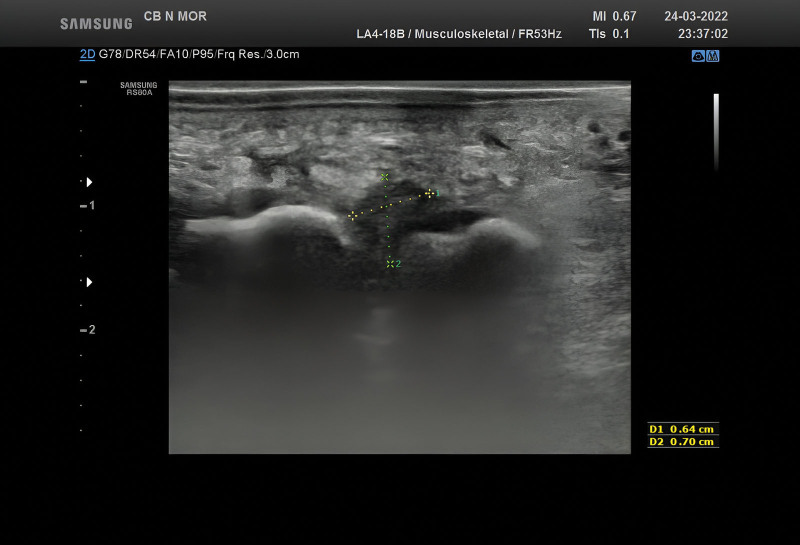
A distinct, well-defined picture measuring 6/7 mm in size, located at the ends of metatarsals III and IV. This image appears as a nodular, hypoechoic, and homogeneous structure, and there is no Doppler signal detected.

The use of imaging diagnostic procedures is limited to unequivocal situations that can’t be confirmed through clinical examination alone, as well as cases where there are suspected multiple and/or multifocal lesions.^[2,3,6]^ Additionally, imaging techniques are used to differentiate between various clinical disorders that may mimic Morton neuroma.^[3,4]^ Plain radiographs are not specific; however, they can be utilized to exclude local fractures, Freiberg disease, and osteoarthritis.^[2,3,5,7]^ The ultrasonography (US) can be performed to validate the diagnosis, usually when the lesion reaches a size of 5 mm. The typical US appearance is an ovoid hypoechoic mass, parallel to the long axis of the foot^[2]^ (Fig. 2). Although the US is an available diagnostic tool with a 90% sensitivity and 88% specificity rate,^[8]^ it is considered an unreliable method, mainly because it is highly operator-dependent.^[6]^ Nevertheless, the US remains the first-line imaging tool to positively diagnose Morton neuroma and differentiate it from other pathologies.^[2,4]^ On the other hand, an magnetic resonance imaging (MRI) can be performed to exclude additional causes of intermetatarsalgia such as metatarsal, adventitial bursitis or synovitis, inflammatory arthritis, plantar plate injury, gout, rheumatoid arthritis, and osteoarthritis.^[5]^ Therefore, MRI has a sensitivity rate of 93%, but its specificity is quite low at 68%.^[3]^ Additionally, only one-third of Morton neuromas detected by MRI are clinically asymptomatic.^[7]^

The utilization of EDX techniques in the diagnosis of Morton neuroma is limited due to the significant range of test results. However, J.M. Pardal-Fernandez and his team developed an electroneurography technique for diagnosing Morton neuroma.^[9]^ Orthodromic nerve conduction study was conducted on the lateral and medial plantar nerves. Ring electrodes were positioned in each intermetatarsal region to provide stimulation. Two recording electrodes were positioned using subdermal needles at the inferior border of the tibial malleolus. To conclude, the research team compared the nerve conduction velocity of the subjects with that of the control group. The study identified a specific threshold for conduction velocity, which indicated a sensitivity of 82% and a specificity of 85%.^[9]^

## 4. Tarsal tunnel syndrome

### 4.1. Introduction

Tarsal tunnel syndrome (TTS) is a focal entrapment neuropathy in which the tibial nerve or its branches are compressed inside the tarsal tunnel, located on the inner side of the ankle. The clinical manifestation of patients fluctuates based on the site of compression and is defined by classical symptoms of peripheral nerve entrapment: pain, paresthesia, tingling, and/or sensory loss.^[10]^ The exact prevalence of TTS remains unknown, however, it seems to be more common in women and the adult population. Moreover, it has been reported that the probability of this condition is higher among athletes compared to the general population.^[11]^

### 4.2. Anatomy, etiology, and pathophysiology

**Figure 3.**
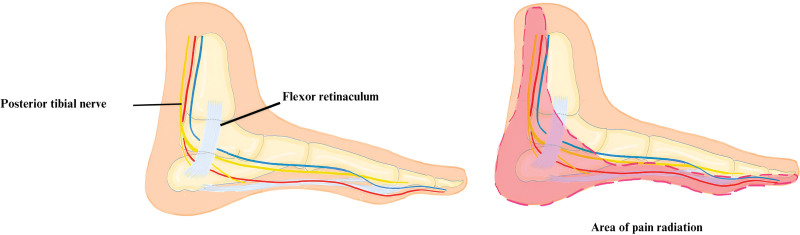
Panel A illustrates a detailed medial view of the tarsal tunnel, showing the anatomical elements included inside it, such as the tibial nerve, arteries, and tendons. These features are crucial for comprehending the pathophysiology of tarsal tunnel syndrome. Panel B indicates the typical area of pain radiation associated with TTS. TTS = tarsal tunnel syndrome.

The tarsal tunnel is an osteofibrous structure situated on the medial side of the ankle. The floor of the structure is composed of multiple bones, including the distal tibia, calcaneus, and talus. Meanwhile, it is covered by the fibrous flexor retinaculum.^[12]^ The canal contains the tendons of the tibialis posterior, flexus hallucis longus, and flexor digitorum longus muscles.^[10]^ Additionally, it includes the posterior tibial artery and vein, as well as the posterior tibial nerve, which descends through the posterior leg^[11]^ (Fig. 3). Upon entering the tarsal tunnel, the tibial nerve divides into a medial calcaneal nerve and then splits into lateral and medial plantar nerves.^[10,11]^

The pathophysiology of TTS can be understood by considering the natural features of the tarsal tunnel, which is a restricted anatomical structure.^[11]^ Therefore, any reduction in the tunnel’s volume from outside factors or any augmentation in the volume of the structures inside the tunnel can exert pressure on the nerve structures.^[10,12]^ The predominant etiological causes and/or risk factors that contribute to the development of TTS can be categorized as: (a) direct trauma: compression or bony lesions apply the pressure inside the tunnel or may contribute to a secondary hemorrhage and adhesions a consequence^[13]^; (b) vascular disorders: varicose veins, arterial and venous malformations^[10]^; (c) soft tissue inflammation: osteoarthritis, rheumatoid arthritis, and flexor tendinitis^[10,13]^; (d) space occupying lesions: osteophytes, osteochondromas, peripheral nerve tumors, ganglionic cysts, perineural fibrosis, hypertrophied muscles, and hypertrophy of flexor retinaculum^[10,11,13]^; (e) edema resulting from an excessive level of fluid^[11]^ or myxedema in hypothyroidism^[10]^; (f) obesity and diabetes^[11,13]^; (g) repetitive movements of the foot: specifically, plantarflexion and eversion of the foot, which anatomically reduces the space within the tarsal tunnel^[10]^; (h) iatrogenic factors: occurring after surgery or arthroscopic procedures^[10]^; (i) presence of extra and/or accessory muscles around the ankle (such as the flexor digitorum accessorius longus muscle)^[10,13]^; and (j) idiopathic causes, accounting for up to 40% of cases.^[10,11,14]^

### 4.3. Clinical presentation and physical examination

The clinical presentation of patients can vary depending on the site and specific branches of the tibial nerve that are being compressed.^[10]^ A common presentation of a patient with TTS includes intense and imprecise sensations such as pain, tingling, hyperesthesia, burning, and numbness.^[10,11]^ These symptoms mostly affect the heel and medial ankle and are particularly pronounced in the sole.^[10]^ The Valleix phenomenon refers to the occurrence of severe and persistent paresthesia that often radiates towards the calf and leg.^[11,14]^ The symptoms typically worsen during physical activity^[10]^ and are more pronounced at night.^[11]^ The onset is insidious and frequently occurs unilaterally.^[11,14]^ Furthermore, during the examination, clinicians can induce local or radiating paresthesia by tapping on the medial ankle.^[11]^ Nevertheless, the Tinel sign exhibits a sensitivity of 58% and may be positive in patients with polyneuropathy or in healthy individuals.^[15]^ Hence, it is necessary to take into consideration other provocative maneuvers as well. The most prevalent test is the dorsiflexion-eversion test, a procedure that generates pain and/or numbness in the ankle or sole by passively dorsiflexing and everting the ankle for 5 to 10 seconds.^[10,11,15]^ The sensitivity of this test is 98%, and the specificity rate is 86%.^[15]^ Additionally, the Trepman test involves patients reporting pain and/or numbness in the ankle or sole after passive plantarflexion and inversion at the ankle.^[10,11,15]^ Finally, the triple compression test combines elements of the Trepman and Tinel tests.^[10,15]^ This combination results in sensitivity and specificity scores of 100%.^[15]^

### 4.4. Electrodiagnostic and imaging techniques

The topic of the diagnostic nerve conduction study (NCS) for TTS is a subject of debate.^[11,16]^ Due to the absence of a definitive gold standard for diagnosing TTS,^[13]^ there is a lack of qualitatively reliable data about the sensitivity and specificity of EDX.^[11,13]^ Thus, certain research has demonstrated that NCS produces a significant proportion of false negative outcomes, whereas EMG reveals a notable incidence of false positive results ranging from 10% to 43%.^[16]^ Nevertheless, following defined recommendations, prolonged latencies of the MPN and LPN at the ankle^[17]^ and/or reduced amplitude compound muscle action potentials of the MPN and LPN—when measured at the level of abductor hallucis brevis and, respectively, abductor digiti quinti pedis muscles—are suggestive for the diagnosis of TTS.^[11]^ However, due to the considerable rate of inaccurate measurements, it is advisable to consistently compare the results with the asymptomatic side.^[13]^

**Figure 4.**
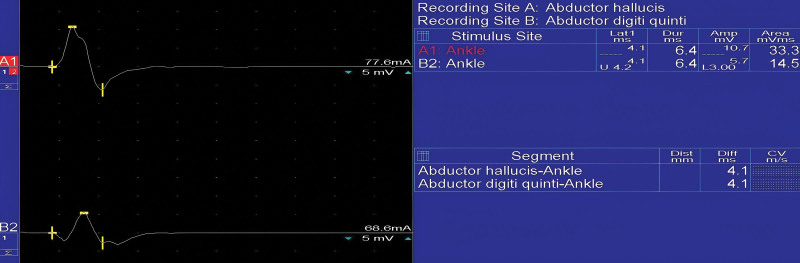
Motor nerve conduction study on the medial and lateral palmar nerve. Stimulation is performed at the level of the medial malleolus and the motor response is recorded on the abductor hallucis brevis and abductor digiti quinti muscles.

D.C. Preston and B.E. Shapiro provided a comprehensive description of the recommended NCS method for TTS, which includes several different examinations (A–D). Each study involves a recording electrode (G1), reference electrode (G2), ground and stimulation site (S1). For the medial plantar motor study (A), the recording site is set at the level of the abductor hallucis brevis muscle, with the G1 electrode placed closer to the body muscle and 1 cm below the navicular prominence, while G2 is positioned above the metatarsophalangeal joint of the hallucis. Nerve stimulation is conducted at the medial ankle, slightly closer and towards the back of the medial malleolus. The recording site for the lateral plantar motor study (B) is situated at abductor digiti quinti pedis, with G1 positioned at the midpoint between the lateral sole and the lower edge of the lateral malleolus. The G2 is positioned directly above the metatarsophalangeal joint of the little toe. The stimulation site is located at the medial part of the ankle, slightly proximal and posterior to the medial malleolus.^[18]^ The morphology of compound muscle action potential in a healthy patient can be recognized in Figure 4.

**Figure 5.**
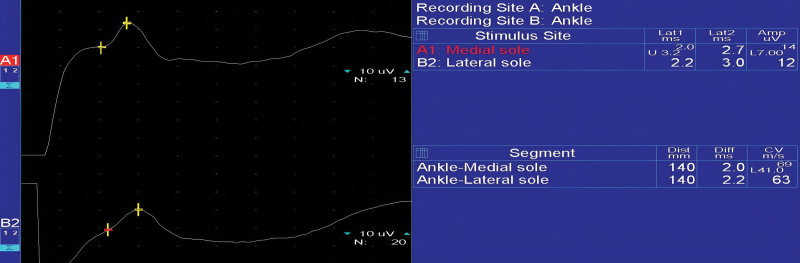
The medial and lateral plantar mixed study. The recording electrode is placed at the ankle, while nerve stimulation is conducted at the medial sole, along the area of the medial and lateral plantar nerves.

In the medial plantar mixed study (C), G1 is situated proximal and posterior to the medial malleolus, while G2 is positioned 3 to 4 cm proximal to G1. S1 is located at the medial sole in the area of MPN, approximately 14 cm from the recording electrodes. The lateral plantar mixed examination (D) is similar to the medial plantar mixed study in terms of G1 and G2, being identical. S1 is found at the medial sole in the area of LPN, about 14 cm away from the recording electrodes.^[18]^ The NCS of MPN and LPN can be seen in Figure 5.

Nevertheless, the patient’s medical history and the anamnesis play a major role in the diagnosis.^[19]^ Therefore, physicians may exclude several risk factors that can mimic the symptoms, such as hypothyroidism, mixedem, or a history of diabetes.^[10,15,19]^ Alternatively, they may identify pathological foot deformities through an extensive physical examination.^[13,19]^ However, the utilization of imaging techniques is necessary to provide confirmation.^[19]^ Thus, the US remains the primary method, while the superficial location of the tarsal tunnel makes it accessible.^[14,19]^ The presence of hypoechogenity, fascicular structure, and absence of echogenic fat around the nerve are suggestive for the diagnosis of neuropathy.^[20]^ The Doppler method increases US practicality in the identification of vascular problems, such as varices or vascular malformations.^[19]^ Furthermore, the US offers several advantages: it is lower in cost, easily accessible, requires less time for examination, and provides high-quality images.^[14,19]^

However, MRI continues to be the most reliable method for diagnosing space-occupying lesions.^[11,20]^ Additionally, it is possible to determine the degree of the flexor retinaculum thickness or demonstrate the compression of the nerve caused by trauma, fracture, and fibrosis of perineural structures.^[11,20]^ In subacute cases of entrapment, the presence of muscular edema indicates the probability of the diagnosis.^[20]^ However, it is important to differentiate it from additional causes such as dermatomyositis, infectious, inflammatory and traumatic myositis, radiotherapy, compartment syndrome, and rhabdomyolysis. Plantar fasciitis is a possible diagnosis that needs to be eliminated as an option.^[14,20]^ Performing bilateral CT scans or plain radiographs can be useful in examining the skeletal structure and identifying the existence of osteophytes, fractures, or pathological foot deformities like hindfoot valgus and varus.^[11,19,20]^

### 4.5. Baxter neuropathy

Baxter neuropathy, also known as inferior calcaneal neuropathy, involves the entrapment of the first branch of the lateral plantar nerve, often leading to chronic heel pain. This condition is frequently misdiagnosed as plantar fasciitis due to overlapping symptoms, which include pain and tenderness in the medial and plantar aspect of the heel.^[21]^ Ultrasound-guided nerve blocks have emerged as a valuable tool in both the diagnosis and treatment of Baxter neuropathy. This technique allows for precise localization and treatment of nerve entrapment, providing significant pain relief and aiding in the recovery process.^[22]^ MRI findings typically show an abnormal signal intensity around the nerve, which is indicative of nerve compression. Conservative management includes physical therapy and corticosteroid injections, while surgical intervention is considered when conservative measures fail.^[23]^

## 5. Obturator nerve entrapment syndrome

### 5.1. Introduction

Obturator nerve entrapment, also known as obturator tunnel syndrome, is a medical condition that causes pain in the pelvic or groin region.^[24]^ Thereby, the presence of different structures passing through these regions makes the differential diagnosis challenging. Moreover, multiple factors contribute to this condition, including disorders related to muscles, tendons, bones, joints, blood vessels, and nerves.^[25,26]^

### 5.2. Anatomy, etiology, and pathophysiology

**Figure 6.**
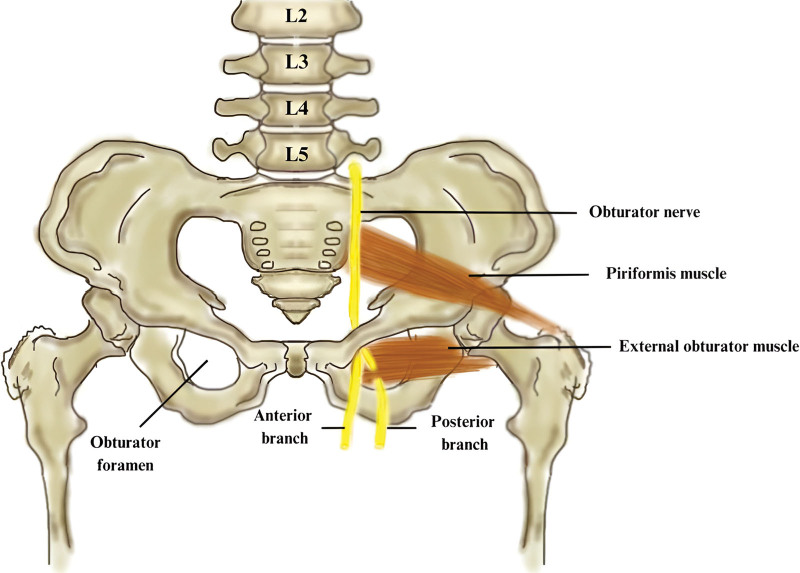
This figure displays the trajectory of the obturator nerve, which originates from the lumbar plexus and traverses the obturator foramen to provide innervation to the medial part of the thigh.

The obturator nerve (ON) is formed by the anterior divisions of the ventral rami of the L2–L4 fibers.^[5]^ After its emergence, the nerve passes down through the psoas major muscle and reaches the pelvis.^[25]^ At this level, it is situated medial to the femoral nerve and together with the obturator artery, leaves the pelvis through the obturator foramen.^[5,18,24]^ In 50% of situations, it divides into an anterior and posterior branch situated on the anterior and posterior sides of the external obturator muscle^[27]^ (Fig. 6). In the rest of the cases, the separation occurs in the pelvis or thigh. Likewise, an accessory ON has also been identified.^[28]^

The anterior branch of the obturator nerve provides innervation to the adductor brevis muscle, which is the only muscle exclusively innervated by this nerve. It also innervates the adductor longus, gracilis, and sometimes the pectineus muscle, however, the primary innervation for the pectineus muscle comes from the femoral nerve.^[5,27]^ The posterior branch innervates the obturator externus, adductor magnus, and brevis muscles.^[5]^ The anterior branch for the anteromedial hip joint and medial thigh, as well as the posterior branch for the knee joint, provide the sensory distribution of the ON.^[5,24]^ Nevertheless, the innervation and trajectory of the nerve present significant anatomical variability among individuals, resulting in different diagnostic and therapeutic implications.^[26]^ Therefore, the main site for entrapment occurs at the anterior branch, which is surrounded by a fascial layer that contains adipose and connective tissue, as well as the vascular pedicle of the medial femoral circumflex artery.^[27]^ A thick intramuscular septum^[27]^ or different conditions within the obturator canal,^[5]^ are examples of another etiology. Furthermore, after total hip replacement surgeries, ON injuries lead to persistent discomfort in the groin area, associated with adductor muscle weakness.^[24,28]^ Furthermore, the removal of cement from the arthroplasty could play a role in the occurrence of ON entrapment.^[26]^ In addition, the lithotomy posture, commonly used in urologic and gynecologic procedures, has been linked to nerve injury when combined with abdominal surgery.^[29]^

However, with all these etiologies and other causes such as pelvic fractures, neoplasms and hernias, which typically have a larger impact on pelvic nerves,^[25,28]^ isolated ON injury, though entrapment, occurs rarely.^[24]^

### 5.3. Clinical presentation and physical examination

Male patients tend to have compression of the obturator nerve, which is associated with practicing sports that include kicking and lateral movements, such as football and rugby.^[24,25]^ This phenomenon may also be attributed to physical differences, such as the reduced transverse pelvic inlet and a decreased subpubic angle observed in males.^[28]^ Exercise-induced pain in the groin, pelvic region, or medial thigh is something that patients can report. The discomfort is profound and diffuse, starting from the adductor muscle origin and migrating to the medial thigh, medial knee, or ipsilateral anterior superior iliac spine.^[26,27]^ This condition gets worse when the hip is extended, abducted, and internally rotated against resistance.^[24,26,28]^ Moreover, anesthesia and paresthesia may be additional symptoms in this region area. Furthermore, anesthesia and paresthesia may manifest as extra symptoms in this region.^[26,27]^ Adductor muscle weakness, described as a “lack of propulsion during running”^[29]^ associated with spasms at this level, is consistently present.^[27]^ A sensation of tenderness during deep palpation at the adductor canal^[24,28]^ and the absence of the adductor reflex on the same side could indicate the presence of this medical condition.^[27,28]^ Some evidence, such as pectineal muscle stretch and Howship-Romberg sign, is pain-eliciting maneuvers that may support the diagnosis but are not pathognomonic.^[27]^ The groin pain related to ON entrapment must also be differentiated from other disorders such as adductor tendinopathy, avascular necrosis, greater trochanter bursitis, coxarthrosis, obturator hernia, stress fracture and enthesopathy. These conditions are commonly reported in professional athletes^[24,26–28]^

### 5.4. Electrodiagnostic and imaging techniques

During the ON assessment, NCS cannot be performed to assess the nerve entrapment.^[28]^ In contrast, the EMG of the thigh adductors is useful for evaluating nerve function. At the thigh, adductor muscles (gracilis, adductor longus and adductor magnus) function as a unit, therefore, they will be examined together.^[18]^ The patient is sitting on the affected side, with the thigh and knee in a flexed position. To initiate movement, we ask the subject to internally rotate the hip.^[15]^ A longer needle of 37 or 50 mm is inserted on the medial side of the thigh, around 4 fingerbreadths distal to the pubis. Therefore, in cases of acute denervation, we may detect fibrillation, respectively, motor unit potential characterized by high amplitude and duration in cases of chronic reinnervation.^[27]^ Furthermore, additional investigations aid to the diagnosis. Ultrasound can be used to identify nerve edema and muscle atrophy, while STIR or FLAIR MRI imaging can detect muscle denervation with high sensitivity.^[5,27,28]^

## 6. Superior gluteal nerve entrapment

### 6.1. Introduction

The entrapment of the superior gluteal nerve may occur when the piriformis muscle compresses it, leading to weakening in the affected hip and tenderness when pressure is applied to the upper outside portion of the buttocks, just above the greater sciatic notch.^[30]^ Moreover, superior gluteal nerve entrapment is a frequently misdiagnosed cause of buttock pain.^[31]^

### 6.2. Anatomy, etiology, and pathophysiology

**Figure 7.**
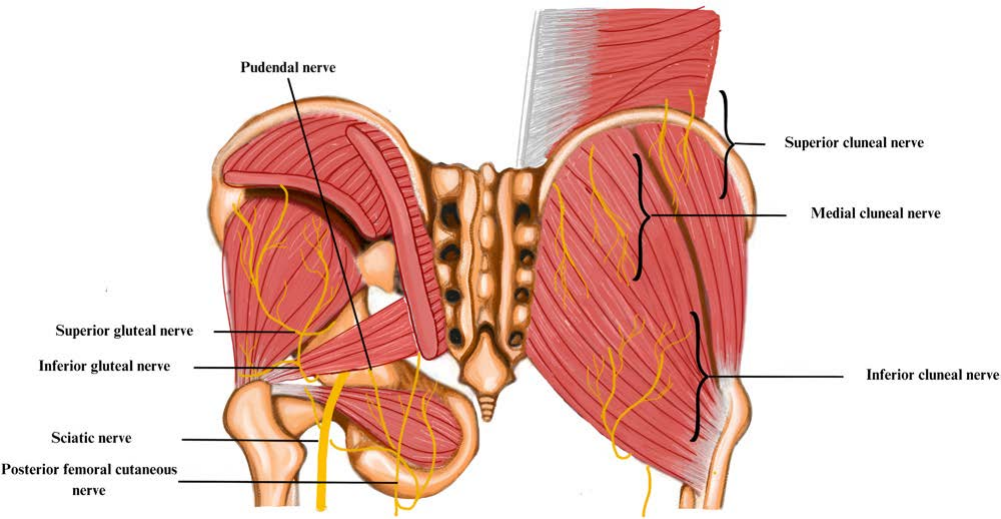
The image illustrates the lumbar and sacral regions, displaying the complex structure of the cluneal and gluteal nerves. The lumbar and sacral plexuses (L1–S4) separate into the superior, middle, and inferior cluneal nerves, each following distinct paths around the iliac crest and through the thoracolumbar fascia. Simultaneously, the gluteal nerves, which originate from the sacral plexus, can be identified by their upper and lower branches that pass above and below the piriformis muscle.

The superior gluteal nerve (SGN) arises from the sacral plexus, from the posterior divisions of the L4, L5, and S1 spinal roots. It passes through the greater sciatic notch, between the gluteus medius muscle (GMM) and the superior edge of the PM (Fig. 7). The SGN is a mixed nerve, accompanied by the superior gluteal artery and vein. The sensory innervation is restricted exclusively to the greater trochanter of the femoral neck. The motor distribution includes the abductor muscles of the thigh. The GMM receives innervation from the superior branch, while the inferior branch innervates the gluteus medius, gluteus minimus, and tensor fascia lata muscles.^[31]^ Iatrogenic factors, including the posture of the patient with hip flexion and adduction during surgery, are the primary cause of SGN entrapment.^[32]^ Common causes of nerve damage include trauma on the nerve from falling on the buttock, injuries from motor vehicle accidents, excessive rotation of the leg or hip, and incorrect technique during intramuscular injections, involving the development of a hematoma.^[31]^ The biomechanical conditions indicate that an elevated tension of the piriformis muscle, caused by lumbar hyperlordosis and hip flexion, leads to morphostatic conditions that can account for the increased pain reported during walking upwards or climbing stairs.^[32]^

### 6.3. Clinical presentation and physical examination

Patients commonly report discomfort in the buttocks and lower back that radiates to the popliteal fossa and is exacerbated by internal rotation. This pain usually occurs during prolonged sitting or standing, as well as while walking or running. As a result, the patients will adopt a posture that reduces discomfort by sitting on the opposite buttocks or crossing their legs. Furthermore if the weakness of the abductor muscles is evident, patients will present the Trendelenburg sign.^[31]^ Therefore, SGN entrapment is characterized by 3 specific symptoms: (a) pain in the buttocks; (b) weakness in the hip abductors resulting in a particular swayed walk; and (c) tenderness on the lateral side of the sciatic notch.^[30]^ Moreover, to the differential diagnosis with other sacroiliac pathologies or myofascial muscle conditions, a pain-inducing maneuver is executed with the patient in a standing position, with the hips flexed. Thus, the examiner applies pressure to the posterior superior iliac spine and greater sciatic notch, laterally and superiorly between the piriformis and GMM.^[31]^ Simultaneously, other possible causes for buttock pain can be investigated such as: lumbar spine disorders, sacroiliac pathology, piriformis or gluteus medius spasm, sciatic entrapment, and posterior femoral cutaneous nerve.^[31]^

### 6.4. Electrodiagnostic and imaging techniques

During the SGN assessment, electromyography (EMG) is conducted to analyze the activity of 2 specific muscles that are innervated by the nerve. In the GMM evaluation, the patient adopts a lateral position, with the affected side oriented upwards. The needle electrode is placed into the lateral side of the thigh, about 2 to 3 fingerbreadths distal of the iliac crest. To activate, the patient is instructed to abduct the thigh. The assessment of the tensor fascia lata muscle is conducted with the patient maintaining the same position as in the prior test. For activation, the patient is instructed to raise the ankle, consequently executing an internal rotation of the thigh.^[18]^

Additional useful diagnostic investigations involve the use of US examination to detect piriformis spasm, as well as the application of ultrasound-guided anesthetic injections.^[31]^ X-rays are not performed, nevertheless, MRI can be used to assess the trajectory of the SGN and detect any denervation alterations in the muscles innervated by the SGN using coronal and sagittal images.^[31,33]^

## 7. Cluneal nerve entrapment syndrome

### 7.1. Introduction

#### 7.1.1. Superior and middle cluneal nerve entrapment

Low back pain (LBP) is a common health complaint. The cause of this medical condition is generally difficult to determine, and the diagnostic attention is primarily focused on the intervertebral disc, lumbar facet joints, and sacroiliac joint.^[34]^ However, recent studies have focused on peripheral nerve entrapment, including the compression of the superior or middle cluneal nerve (SCN or middle cluneal nerve [MCN]), but only a few cases can be attributed to this type of nerve entrapment.^[35]^

Entrapment of SCN is a complex problem in cases of LBP and may lead to groin pain and/or leg symptoms in 57% of patients.^[36]^ In a prospective analysis, the authors found that 14% of patients with LBP reported entrapment of the SCN. Furthermore, the occurrence of bilateral SCN entrapment was found to be between 20% and 33%, with females representing 55% to 63% of all cases. Although the average age of onset often ranges between 55 and 68 years, cases of SCN entrapment have also been observed in younger individuals. There is a lack of comprehensive information on the occurrence of MCN entrapment, but current data indicates that it exhibits similarities with SCN.^[34]^

#### 7.1.2. Inferior cluneal nerve entrapment

Neuropathic ischial pain, often confused with sciatic neuropathy, can occasionally involve areas beyond the typical innervation of the sciatic nerve. Pain resulting from inferior cluneal nerve entrapment (ICN) can be reported in the caudal and medial parts of the buttocks, the upper region of the posterior part of the thigh, and may also extend to the scrotum or labia majora.^[37]^

### 7.2. Anatomy, etiology, and pathophysiology

#### 7.2.1. Superior and middle cluneal nerve entrapment

The SCN and MCN are exclusively sensory cutaneous nerves, with their distribution territories in the lumbar region and buttocks. The SCN emerges from the lower thoracic and lumbar posterior nerve roots (T11, L5). It follows a trajectory from superior-medial to inferior-lateral and penetrates the thoracolumbar fascia at the level of the iliac crest.^[35]^ The nerves provide sensory innervation to the skin over the 2/3 upper of the gluteal muscles and present a complex and diverse pattern of branching. Furthermore, in the majority of cases, there is at least 1 SCN nerve passing through the osteofibrous tunnel^[34]^ (Fig. 7). This level represents a possible ongoing entrapment as the nerve permeates the posterior sacroiliac ligament, situated 5 to 9 cm away from the midline.^[38]^ Furthermore, the SCN and MCN can become trapped around the iliac crest.^[36]^ Therefore, several conditions that increase muscle tone, for example, vertebral body fracture, or induce stretching, increase the risk of SCN entrapment. Moreover, root nerve irritation or a herniated disc could also contribute to the “double crush” phenomenon.^[34]^

#### 7.2.2. Inferior cluneal nerve entrapment

The ICN originates from the posterior femoral cutaneous nerve of the thigh and includes sensory branches from the S1 to S3 roots. The nerve trajectory follows a parallel path to the sciatic and pudendal nerves as it passes through the sciatic notch. The entrapment site usually occurs in 2 specific locations: (a) where the perineal ramus passes under the ischium towards the perineum, causing nerve compression due to sitting or thigh rotation, and (b) proximal at the intersection of the piriformis and sciatic spine, where the roots of the posterior femoral cutaneous nerve are susceptible to compression.^[37]^

### 7.3. Clinical presentation and physical examination

#### 7.3.1. Superior and middle cluneal nerve entrapment

Typically, the patients complain of LBP and discomfort in the buttocks. The symptoms intensify with lumbar movement and may mimic radiculopathy, which includes tingling sensations radiating down the leg and intermittent claudication.^[35]^ In addition, some authors suggest multiple diagnostic criteria for the involvement of SCN and MCN. (a) LBP involving the buttocks and iliac crest; (b) symptoms exacerbated by movement; (c) Tinel-like sign at the site of nerve penetration; (d) numbness and radiating pain from the area when pressure is applied to trigger sites; and (e) relief of symptoms through the use of anesthetic blocks at the trigger points.^[34]^ The lower back pain and buttock pain can be attributed to other potential causes, like facet or sacroiliac arthropathy, piriformis syndrome symptoms, or myofascial inflammation.^[34]^

#### 7.3.2. Inferior cluneal nerve entrapment

Patients with ICN entrapment commonly report sensations of burning, tingling, or numbness in several particular areas: (a) inferior and medial buttocks; (b) dorsal and proximal thigh; and (c) the lateral edge of the anus and the skin of the scrotum or labia majora. These symptoms worsen when sitting on hard surfaces, such as chairs or bicycle seats. Usually, soft tissue palpation reveals non-radiating pain, which becomes more intense when applying deep pressure over the sciatic notch. The absence of motor involvement frequently leads to the misdiagnosis of entrapment as pudendal canal syndrome.^[37]^

### 7.4. Electrodiagnostic and imaging techniques

The EDX studies are not relevant for diagnosis but are commonly used to rule out other causes of LBP and buttock pain.^[37]^ Standard CT and MRI scans cannot provide relevant information because the nerves are too small to be properly recognized. However, high-resolution CT might help identify the bony groove within the osteofibrous tunnel.^[35]^ Furthermore, the physician may consider plain X-rays to examine for vertebral body compression fractures due to the higher risk of entrapment of the SCN in these patients. In addition, ultrasound is a valuable tool for diagnosing entrapment neuropathies and facilitating diagnostic injections.^[34]^

## 8. Conclusion

Lower limb entrapment neuropathies cover a spectrum of disorders, which include less common conditions such as tarsal tunnel syndrome, Morton neuroma, obturator nerve entrapment syndrome, superior gluteal nerve entrapment, and cluneal nerve entrapment syndrome. These are associated with particular symptoms and physical signs identified through examination and pain-inducing maneuvers, which should be taken into consideration in atypical presentations of radiculopathies, plexopathies, orthopedic conditions, or gynecological pathologies. Thus, this review holds significance in situations where the patient’s symptoms and clinical assessment diverge from the standard routine examination process. In this case, the examiner must be aware of the existence of these unusual conditions, consider them, and search for appropriate diagnostic solutions.

EDX measurements are the primary examinations used to determine if nerve entrapment is present. A specialist with extensive experience is necessary for this investigation, as entrapment neuropathies could result in contradictory findings if not properly conducted. In situations where EDX findings are unclear or there is suspicion of an additional entrapment cause, the use of MRN, MRI, or ultrasound becomes necessary. In addition, ultrasonography or CT-guided injections of lidocaine at the nerve location can provide symptomatic relief and also provide diagnostic and prognostic value. Furthermore, since recent progress in noninvasive techniques, such as endoscopic decompression, disorders like these can now be managed efficiently. Thus, this review underscores the medical importance of identifying these specific conditions by highlighting the need for precise diagnosis through the use of suitable investigation methods.

## Acknowledgments

We express our sincere thanks to Dr Corina Bocsa for generously sharing the ultrasound images.

## Author contributions

**Conceptualization:** Nicu Cătălin Drăghici, Roxana Bolchis.

**Methodology:** Nicu Cătălin Drăghici, Roxana Bolchis.

**Project administration:** Dafin Fior Mureșanu.

**Software:** Diana Maria Domnița.

**Supervision:** Livia Livinț Popa, Dafin Fior Mureșanu.

**Validation:** Vitalie Văcăraș.

**Visualization:** Silvina Iluț.

**Writing – original draft:** Roxana Bolchis, Atamyrat Bashimov.

**Writing – review & editing:** Nicu Cătălin Drăghici, Hanna Maria Dragoș, Irina Vlad.
